# H-FABP: A new biomarker to differentiate between CT-positive and CT-negative patients with mild traumatic brain injury

**DOI:** 10.1371/journal.pone.0175572

**Published:** 2017-04-18

**Authors:** Linnéa Lagerstedt, Juan José Egea-Guerrero, Alejandro Bustamante, Joan Montaner, Ana Rodríguez-Rodríguez, Amir El Rahal, Natacha Turck, Manuel Quintana, Roser García-Armengol, Carmen Melinda Prica, Elisabeth Andereggen, Lara Rinaldi, Asita Sarrafzadeh, Karl Schaller, Jean-Charles Sanchez

**Affiliations:** 1 Department of Human Protein Sciences, Faculty of Medicine, University of Geneva, Geneva, Switzerland; 2 NeuroCritical Care Unit, Virgen del Rocío University Hospital, Seville, Spain; 3 Neurovascular Research Laboratory, Vall d’Hebron Institute of Research (VHIR), Universitat Autònoma de Barcelona, Barcelona, Spain; 4 Division of Neurosurgery, Geneva Neuroscience Center, Department of Clinical Neurosciences, Geneva University Hospitals, Geneva, Switzerland; 5 Intensive Medicine Unit, Hospital Universitario La Paz, idiPAZ, Department of Medicine, Universidad Autónoma de Madrid, Madrid, Spain; 6 Neurosurgical department, Neuroscience Unit, Hospital Universitari Germans Trias i Pujol, Badalona, Spain; 7 Emergency Department, Hospital de Tortosa Verge de la Cinta, Tortosa, Spain; 8 Emergency Center, Geneva University Hospitals, Geneva, Switzerland; 9 Department of Surgery, Geneva University Hospitals, Geneva, Switzerland; 10 Department of Neurosurgery, University Hospital Heidelberg, Heidelberg, Germany; University of Florida, UNITED STATES

## Abstract

The majority of patients with mild traumatic brain injury (mTBI) will have normal Glasgow coma scale (GCS) of 15. Furthermore, only 5%–8% of them will be CT-positive for an mTBI. Having a useful biomarker would help clinicians evaluate a patient’s risk of developing intracranial lesions. The S100B protein is currently the most studied and promising biomarker for this purpose. Heart fatty-acid binding protein (H-FABP) has been highlighted in brain injury models and investigated as a biomarker for stroke and severe TBI, for example. Here, we evaluate the performances of S100B and H-FABP for differentiating between CT-positive and CT-negative patients. A total of 261 patients with a GCS score of 15 and at least one clinical symptom of mTBI were recruited at three different European sites. Blood samples from 172 of them were collected ≤ 6 h after trauma. Patients underwent a CT scan and were dichotomised into CT-positive and CT-negative groups for statistical analyses. H-FABP and S100B levels were measured using commercial kits, and their capacities to detect all CT-positive scans were evaluated, with sensitivity set to 100%. For patients recruited ≤ 6 h after trauma, the CT-positive group demonstrated significantly higher levels of both H-FABP (p = 0.004) and S100B (p = 0.003) than the CT-negative group. At 100% sensitivity, specificity reached 6% (95% CI 2.8–10.7) for S100B and 29% (95% CI 21.4–37.1) for H-FABP. Similar results were obtained when including all the patients recruited, i.e. hospital arrival within 24 h of trauma onset. H-FABP out-performed S100B and thus seems to be an interesting protein for detecting all CT-positive mTBI patients with a GCS score of 15 and at least one clinical symptom.

## Introduction

Mild traumatic brain injury (mTBI) is common worldwide, with an annual incidence estimated to be above 600/100,000 individuals.[1] Clinicians diagnose and distinguish mTBI patients at risk of intracranial lesions using the Glasgow coma scale (GCS) and clinical symptoms such as headache, nausea, vomiting, loss of consciousness and amnesia.[2,3] The GCS is used to estimate the conscious state and patients scoring between 13 and 15 are classified as having mTBI; indeed the majority of mTBI cases have the GCS best response score of 15.[3] Subsequently, CT scans are often performed to exclude or confirm the existence of brain lesions due to the trauma. However, CT scans are harmful to patients, costly and only a few patients will actually have a brain lesion (5%–8%).[3,4] Developing decision rules for safely distinguishing between patients who will turn out to be CT-positive and CT-negative could help to avoid many unnecessary CT scans.

Several guidelines have put forward decision rules for handling mTBI patients, based primarily on the GCS and risk factors (e.g. clinical symptoms).[3,5] However, this still means that every patient with a symptom will probably undergo a CT-scan. Another approach, which has been widely studied, is the use of blood-based biomarkers. The individual performances of GFAP, Tau and UCHL-1, among others, have all been investigated, though S100B remains the most-studied protein biomarker.[6–16] Moreover, to avoid CT-negative scans, the Scandinavian guideline suggests combining clinical criteria and measuring S100B (cut-off 0.1 μg/L).[5,15] However, the Eastern Association for the Surgery of Trauma guidelines indicate that S100B should not be relied upon for making such a decision.[17] There remains a need to further investigate S100B’s effectiveness (or that of other biomarkers), in conjunction with clinical risk factors, at differentiating between CT-positive and CT-negative patients.

Previous studies on brain injury models have revealed heart fatty-acid binding protein (H-FABP) to be a potentially significant brain injury biomarker.[18,19] It has been shown to be located in the heart but also in the brain and described as an interesting diagnostic marker in stroke, Creutzfeldt-Jakob, Alzheimer’s and Parkinson’s diseases, subarachnoid haemorrhage and severe TBI.[18,20–23] In stroke, H-FABP has a rapid increase in concentration with a peak at 3h after symptom onset and thereafter the concentrations remained high for 5 days. [20] The early increase of blood concentration and steadiness in time make this protein a potential interesting tool for brain lesion diagnostics. Furthermore, H-FABP has already been shown to be able to differentiate between controls and mTBI patients.[23] It thus seemed worthwhile to investigate H-FABP’s effectiveness further as a potential diagnostic tool for differentiating between the CT scan results of mTBI patients. Therefore, the aim of this prospective multicentre study was to evaluate and compare the capacities of H-FABP and S100B in discriminating between CT-positive and CT-negative patients with a GCS score of 15 and at least one additional clinical symptom.

## Methods

### Inclusion and exclusion criteria

This study recruited a total of 261 patients at three different European sites: Geneva (Switzerland), Barcelona (Spain) and Seville (Spain). Written informed consent was obtained from all patients, or their legal representatives, prior to inclusion. Children (< 18 years) were included only after written informed consent from a parent or next-of-kind. To participate, patients needed to fulfil several inclusion criteria: diagnosis of mTBI with a GCS score of 15; presence of at least one clinical symptom (loss of consciousness, amnesia, vomiting or nausea, headache or equilibrium disorder); CT scan performed within 24 h of the trauma (where the presence of epidural haemorrhage, subdural haemorrhage, subarachnoid haemorrhage, intracerebral haemorrhage, contusion with haemorrhage, cerebral oedema or skull fracture was classified as CT-positive); blood sample collected at admission; and age above 14 years old. Exclusion criteria were: pregnancy; GCS score below 15 at admission to hospital; absence of clinical symptoms; no head CT scan; and no signed informed consent form. The study was approved by the relevant local ethics committees: Geneva’s Human Research Ethics Committee (CER: 12–194 / NAC 12–074); Barcelona’s Hospital Universitari Vall d’Hebron Ethics Committee (PR_AG_195–2012); and Seville’s Virgen del Rocío University Hospital Institutional Review Board (2012PI/120).

### H-FABP and S100B assays

Upon hospital arrival, each patient had a serum (Seville and Barcelona) or plasma (Geneva) sample withdrawn, centrifuged, aliquoted and stored at -80°C until analysis. Both H-FABP and S100B were analysed according to the manufacturer’s recommendations. For patients recruited in Geneva, H-FABP was measured using a HK402 kit from Hycult (Hycult Biotech, Uden, The Netherlands) with a limit of quantification (LOQ) ranging between 102–25000 pg/mL, and for patients recruited in Spain it was measured using a K151HTD kit from Meso Scale (Meso Scale Diagnostics, Rockville, MD, USA) LOQ 137–100000 pg/mL. For patients recruited in Geneva and Barcelona, S100B was measured using an EZHS100B-33K kit from Millipore (Millipore, Billerica, MA, USA) LOQ 2.7–2000 pg/mL, and in Seville it was measured using an Elecsys 2010 immunoassay system (Roche Diagnostics, Germany) LOQ 0.005–39 μg/L. Results are presented in ng/mL for H-FABP and μg/L for S100B.

### Statistical analysis

The results from the three sites were merged to form one large multicentre study. Given the heterogeneity in the three cohorts, in terms of the samples (serum and plasma) and assays used, the biomarker results were merged by normalisation, using the median or the z-score as correction factors. This provided comparable results. Patients were dichotomised into CT-positive and CT-negative groups for statistical analyses. As S100B and H-FABP data were non-parametrically distributed, as shown by the Kolmogorov-Smirnov test (p < 0.001), the difference between groups was established using non-parametric Mann-Whitney U tests. Fisher’s exact test and the chi-square test were used to identify significant differences in clinical data between the CT groups, and Spearman rank correlation test was used for correlation between continuous data. The results were further stratified by those clinical factors found to be significantly different in the CT-positive and CT-negative groups. IBM SPSS software, version 20.0 (SPSS Inc., Chicago, IL, USA), was used for all the statistical analyses. The proteins’ diagnostic performances were tested using receiver operating characteristics (ROC) curves with TIBCO Spotfire S+® version 8.2 softeware (TIBCO software Inc., Palo Alto, CA, USA). For each protein, the thresholds were selected at the best cut-off for a sensitivity of 100% and 90%–100%.

## Results

The present study included 261 mTBI patients with a GCS score of 15. Of these, 172 patients came to the hospital ≤ 6 h after trauma, with a mean time (± SD) of 198 min ± 88. The most common clinical symptoms were a loss of consciousness and amnesia (Table 1). A total of 32 patients (19%) were classified as CT-positive, the majority of whom (72%) were men. The most frequent CT-findings were subarachnoid haemorrhage and skull fractures (Table 2). Age was the only clinical variable found to be significantly different between CT-positive and CT-negative patients (p = 0.001) (Table 1). Comparable demographics were also obtained for each cohort separately with only minor differences such as age and the percentage of isolated brain injuries (S1–S3 Tables).

**Table 1:** Characteristics of mTBI patients, ≤ 6 h post-trauma.

	CT-	CT+	p-value^†^
**CT scan**, n (%)	140 (81)	32 (19)	
**Trauma to blood sample** (min)			0.818^‡^
Mean (SD)	199 (86)	194 (96)	** **
Median (min.–max.)	203 (35–360)	208 (40–360)	** **
**Age** (years), mean (SD)	46 (20)	61 (25)	**0.001**^‡^
**Male**, n (%)	101 (72)	23 (72)	0.976
**Symtoms**, y (%)			
Amnesia	84 (60)	24 (75)	0.113
LOC	113 (81)	27 (84)	0.631
Nausea/vomiting	31 (22)	9 (28)	0.47
Headache	59 (42)	8 (25)	0.073
Impaired equilibrium	2 (1)	0 (0)	1
**Mechanism of Injury**, n (%)			
Traffic accident	36 (26)	10 (31)	0.523
Fall	52 (37)	10 (31)	0.531
Assault	25 (18)	5 (16)	0.764
Sports	4 (3)	1 (3)	0.648
Others	19 (14)	6 (19)	0.307
NA	4 (3)		
**Isolated brain trauma**, y (%)	100 (72)	24 (75)	0.727
NA	1 (1)		

^†^ Chi-square test or Fisher’s exact test

^‡^ Mann-Whitney U test

NA: not available

**Table 2:** CT-scan findings detected in CT-positive mTBI patients.

CT-scan findings	Yes, n
Subarachnoid haemorrhage	15
Subdural haemorrhage	9
Intracerebral haemorrhage	4
Epidural haemorrhage	2
Contusion with haemorrhage	9
Edema	1
Skull fracture	15

**Fig 1:**
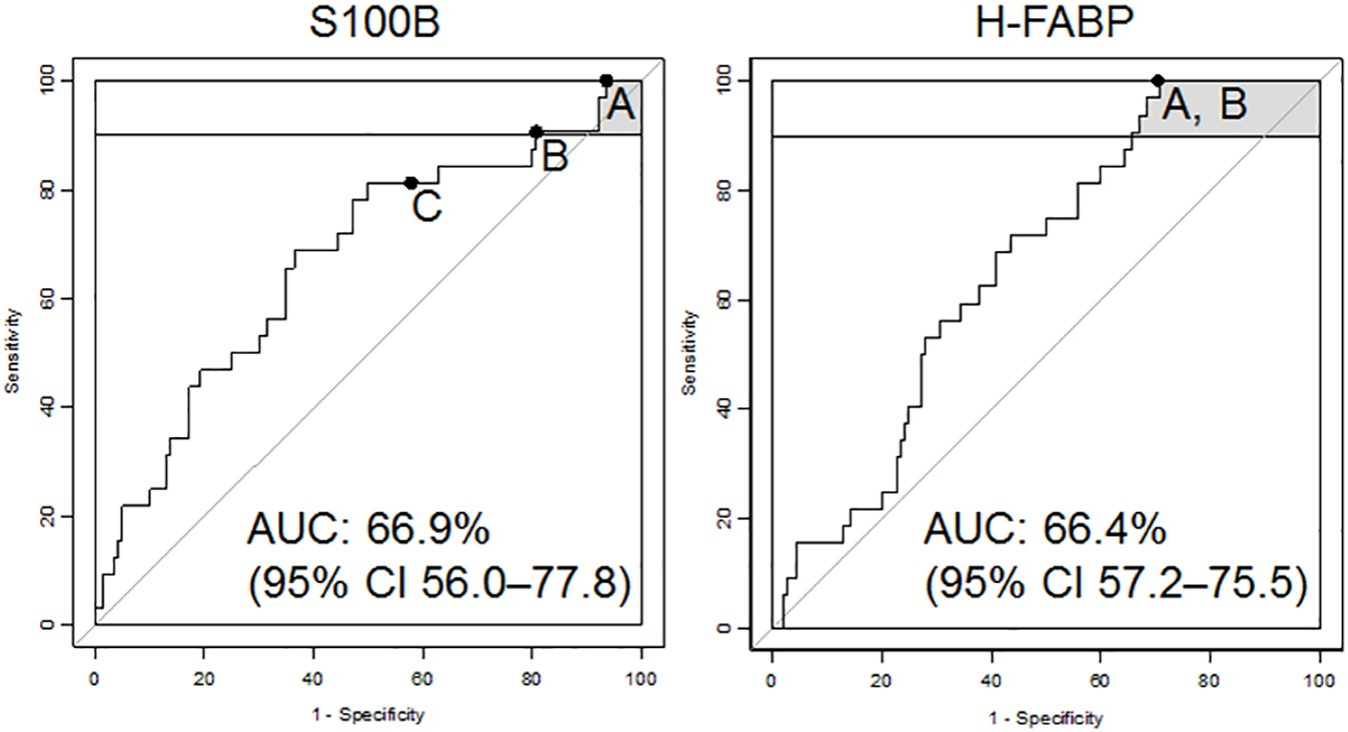
**ROC curves, ≤ 6 h post-trauma, for S100B and H-FABP, representing best performance when set to: a) 100% sensitivity; b) 90%–100% sensitivity; and c) the S100B cut-off 0.1** μ**g/L.**

S100B and H-FABP levels were measured for all patients (at t ≤ 6 h) and showed significantly higher concentrations in CT-positive than in CT-negative patients (p = 0.003 and p = 0.004, respectively). The proteins were also investigated for their individual performances. S100B was first evaluated at the cut-off level of 0.1 μg/L, as previously suggested by Scandinavian guidelines.[5] At this cut-off, S100B showed 42% specificity and 81% sensitivity (Fig 1 and Table 3). With sensitivity set at 100%, S100B displayed a specificity of 6% and H-FABP displayed a specificity of 29%. From a test point of view, investigated using their negative and positive predictive values (NPV and PPV), both proteins displayed an NPV of 100%. For the PPV, H-FABP reached 22%, whereas S100B remained below 20%. The proteins were also evaluated for their best performance when sensitivity was set in the 90%–100% range. In this case, S100B reached 19% specificity, but at the cost of its sensitivity dropping to 91%. There was no observed change in performance for H-FABP (100% sensitivity and 29% specificity).

**Table 3:** Performances when sensitivity was set at 100%, in the 90%–100% range, and when S100B’s cut-off was set at 0.1 μg/L, with corresponding NPV and PPV for patients ≤ 6 h post-trauma.

Protein	Cut-off	SE % (95% CI)	SP % (95% CI)	NPV	PPV
**S100B**	0.042	100 (100–100)	6.4 (2.8–10.7)	100	19.6
	0.071	90.6 (78.1–100)	19.3 (12.9–25.7)	90	20.4
	0.1	81.3 (65.6–93.8)	42.1 (34.3–50.0)	92.6	25.2
**H-FABP**	2.62	100 (100–100)	29.3 (21.4–37.1)	100	22.4

SE, sensitivity; SP, specificity; NPV, negative predictive value; PPV, positive predictive value

The H-FABP and S100B markers were further evaluated in combination. Patients were classified as CT-positive when S100B > 0.042 μg/L and H-FABP > 2.620 ng/mL. Compared to the H-FABP alone, the panel slightly increased specificity, to 30%, with sensitivity remaining at 100%.

CT-positive patients were found to be significantly older than CT-negative patients (p = 0.001) (Table 1). When age was tested as an individual parameter, it reached an AUC of 68%. A sensitivity of 100% could not be attained, indicating that age alone has a low value in identifying CT-positive patients. Significant, though minor, correlations were found between both S100B and age (*r*_*s*_ = 0.171, p = 0.025) and H-FABP and age (*r*_*s*_ = 0.293, p < 0.001).

The Scandinavian guidelines use age ≥ 65 years old as a risk factor.[5] After dichotomising the cohort into < 65 (n = 129) and ≥ 65 years old (n = 43), both S100B and H-FABP showed higher levels in patients ≥ 65 years old than in younger patients (p = 0.004 and p = <0.001, respectively). Marker levels were investigated separately within the younger and older patient groups. In the younger patient group, S100B showed 100% sensitivity and 8% specificity, whereas H-FABP reached 100% sensitivity and 37% specificity. In the older patient group, S100B displayed 100% sensitivity and 32% specificity, compared to H-FABP’s 100% sensitivity and 8% specificity (Table 4).

**Table 4:** The performances of H-FABP and S100B biomarkers at 100% sensitivity, ≤ 6 h post-trauma, dichotomised into younger and older patients.

Age	Protein	CT-, n	CT+, n	Cut-off	SE % (95% CI)	SP % (95% CI)
**< 65**	**S100B**	115	14	0.042	100 (100–100)	7.8 (3.5–13.0)
	**H-FABP**	115	14	2.74	100 (100–100)	36.5 (27.8–45.2)
**≥ 65**	**S100B**	25	18	0.091	100 (100–100)	32.0 (16.0–52.0)
	**H-FABP**	25	18	2.214	100 (100–100)	8.0 (0–20.0)

SE, sensitivity; SP, specificity

The proteins diagnostic performance in patients with only isolated brain trauma was investigated. The cohort population decreased to 100 CT-negative and 24 CT-positive patients. Within this subgroup of mTBI patients, both S100B and H-FABP were significantly increased (p = 0.001) in CT-positive patients. At 100% sensitivity the S100B reached a specificity of 9% whereas the H-FABP increased to 35%. The diagnostic performance was also investigated in the small subpopulation of mTBI patients presenting multiple traumas. None of the proteins had significantly different levels between CT-positive and CT-negative patients and the specificity decreased for both S100B and H-FABP, 5% and 18% respectively, when sensitivity was set to 100% (Table 5).

**Table 5:** The performances of H-FABP and S100B biomarkers at 100% sensitivity, ≤ 6 h post-trauma in mTBI subgroups; isolated brain trauma or multiple trauma patients.

	Protein	CT-, n	CT+, n	Cut-off	SE % (95% CI)	SP % (95% CI)
**Isolated brain trauma**	**S100B**	100	24	0.052	100 (100–100)	9.0 (4.0–15.0)
	**H-FABP**	100	24	2.62	100 (100–100)	35.0 (26.0–44.0)
**Multiple trauma**	**S100B**	39	8	0.031	100 (100–100)	5.1 (0.0–12.8)
	**H-FABP**	39	8	2.829	100 (100–100)	18.0 (7.7–30.8)

SE, sensitivity; SP, specificity

All the results above were based on measurements ≤ 6 h after trauma. However, not all patients seek help immediately and some are transferred from one medical facility to another, increasing the time from trauma onset to blood sampling. The proteins’ predictive performances were therefore also investigated on all the patients who arrived at the hospitals after an mTBI event and within 24 h of trauma onset, with a mean time (± SD) of 250 min ± 172. The number of mTBI patients included here was 261, of whom 41 (16%) were CT-positive (S4 Table). Both H-FABP and S100B levels remained significantly higher in the CT-positive patients (p = 0.006 and p < 0.001, respectively). The proteins’ ability to differentiate CT-positive from CT-negative patients displayed similar results to those described for t ≤ 6 h: for 100% sensitivity, S100B reached 6% specificity and H-FABP reached 26% (S5 Table).

## Discussion

This multicentre study evaluated H-FABP and S100B as potential protein biomarkers for differentiating between CT-positive and CT-negative mTBI patients with GCS scores of 15 and at least one clinical symptom. H-FABP reached 100% sensitivity and 29% specificity, whereas S100B reached 100% sensitivity but only 6% specificity. The proteins’ performances were further investigated when sensitivity was set in the 90%–100% range. Here, S100B reached a specificity of 19% with a sensitivity of 91%. However, H-FABP performed better, with 100% sensitivity and 29% specificity.

In parallel to biomarker research, guidelines are becoming increasingly interested in improving care for mTBI patients based mainly on their GCS scores and risk factors.[3,5] The WHO Collaborating Centre Task Force on mTBI suggested that patients with a GCS of 15 and with one or more risk factors (e.g. age > 60 years old, vomiting, headache or amnesia) should undergo a CT scan.[3] However, these guidelines still mean that there will be many CT-negative results. In the present study, 140 patients were CT-negative (81%), indicating the need for a complementary decision-making aid. Scandinavian guidelines give similar recommendations, but they also suggest using the S100B biomarker with a cut-off level at 0.1 μg/L.[5] Here, this recommended cut-off level only managed 81% sensitivity and 42% specificity, corresponding to 6 false-negative patients.

Whereas S100B has been widely studied as a protein biomarker for diagnosis and decision rules in mTBI patients, H-FABP is relatively unknown in this field. H-FABP is a small (15 kDa) cytoplasmic protein whose primary function is to transport long-chain fatty acids.[23] It was first discovered in the heart, from where it got its name—heart fatty-acid binding protein.[21] It is abundant in the cardiomyocytes and has been studied for many years as a diagnostic tool for different heart conditions.[24,25] This small, intracellular molecule has also been shown to be expressed in the grey matter and neuron cell bodies.[23] H-FABP has therefore been highlighted as a potential brain injury biomarker for several diseases, such as Alzheimer’s, Parkinson’s and stroke, and it has been shown to accurately predict the outcome in severe TBI and be capable of differentiate between mTBI patients and controls.[18,20,22,23,25] The H-FABP has been shown in stroke to have a rapid increase after symptom onset with a peak at 3h and slowly decrease during 5 days.[20] S100B on the other side has a rapid increase however also a rapid decrease in concentration limiting the diagnostic time window.[26] The H-FABP robustness over time makes it interesting as a diagnostic and perhaps also prognostic biomarker. The H-FABP kinetics can be an explanation to the results shown here where patients up to 6h after trauma were included and showed similar results as those included up to 24h after trauma.

S100B is a small (10 kDa), intracellular, calcium binding protein and is found abundantly in astrocytes and adipocytes.[27] Because they are found in different locations, measuring S100B and H-FABP in combination could be interesting. Previous studies in different fields have highlighted the advantages of combining biomarkers into panels to increase their overall performance.[28,29] We therefore combined S100B and H-FABP but used individual 100% sensitivity cut-offs. The sensitivity remained at 100% and the specificity rose to 30%, similar to the level of H-FABP alone. It would be interesting to investigate the differentiating performance of these two molecules and age by using a multivariate analysis. Unfortunately, with regard to the Monte Carlo method, the study population used here was too small.[30]

As different guidelines state, older age can be classified as a risk factor for intracranial lesions.[3,5] Salottolo *et al*. showed that elderly people obtain worse GCS scores than younger patients with the same type of injury severity, which might explain the significantly higher age seen in the CT-positive population than in the CT-negative one.[31] The influence of age on S100B and H-FABP has previously been demonstrated.[21,32–35] The Scandinavian guidelines highlighted that age ≥ 65 could be a risk factor for brain lesions.[5] The present cohort was therefore separated into groups < 65 and ≥ 65 years old. In the younger patient group, H-FABP showed even better performance than for the entire cohort, with sensitivity at 100% and specificity of 37%. In the older patient group, however, the best performances at 100% sensitivity were inversed, with S100B reaching 32% specificity and H-FABP only reaching 8%. These results seem to indicate that H-FABP better discriminates between the CT scan groups among younger patients and S100B does better among older ones. It has previously been shown that H-FABP blood concentration tends to increase in elderly people possibly due to decrease in renal function.[35] This could explain the results shown here, where elderly CT-negative patients will be classified as false positive using H-FABP. Due to the low number of patients in each group, more research is needed to confirm these findings and potentially to create an age ranking table of performances and cut-offs.

The tissue specificity is an important aspect to take under consideration when evaluating a biomarker. Therefore, the differential diagnostic performance of both S100B and H-FABP in mTBI patients with isolated and multiple trauma were evaluated. The capacity of differentiate between CT-positive and CT-negative patients for both H-FABP and S100B decreased to 18% and 5% specificity respectively for 100% sensitivity for patients with multiple traumas due to high blood concentration in CT-negative patients. For mTBI patients with isolated brain trauma the S100B slightly increased to 9% specificity when sensitivity was set to 100%. Interestingly, the H-FABP performance rose to 100% sensitivity and 35% specificity. These results are confirmed by previous studies in severe TBI showing H-FABP to have increased levels in patients with multiple traumas compared to isolated brain traumas.[22]

Another factor that can interfere with biomarker utility is the time between trauma and sample collection. Different earlier articles have used periods of < 3 h to < 6 h for the evaluation of their biomarkers.[11,12,15] The time to hospital arrival has been shown to vary—between the median direct admission time of 1 h and a delayed admission time of 4 h post-trauma—depending on several criteria, such as gender, the influence of alcohol, low energy trauma, and trauma at home or in a public place.[36] The present study’s main results were therefore based on arrival ≤ 6 h post-trauma. However, 89 patients in our cohort arrived later (6–24 h), 9 (10%) of whom were CT-positive, suggesting the need for a decision rule biomarker, even for this patient group. To reflect the real clinical situation as accurately as possible, we included all the patients who arrived at the emergency units within 24 h of trauma onset. These enlarged inclusion criteria raised the cohort size to 261 patients. The results obtained showed similar levels of performance to those observed for arrival ≤ 6 h post-trauma. S100B measurement would only have suggested discharge for 6% of the CT-negative patients while keeping all the CT-positive patients in the hospital; H-FABP measurement would have suggested discharge for 26%. The results coincide with what could be expected seen from previously performed kinetic studies for both S100B and H-FABP. S100B has been shown to rapidly increase but also to decline early.[26] H-FABP’s property of remaining stable over time has been demonstrated previously in stroke patients; the protein’s level increased early and showed highest levels already 3h after symptom onset and elevated levels were maintained for several days.[20] The results suggest that measuring H-FABP for a longer period of time post-trauma could be of benefit to patients arriving a long time after their accident.

Even though this study showed the potential use of H-FABP as a brain injury biomarker for differentiating between CT-positive and CT-negative patients, there were some study limitations. Patients included within this study are a sub-population of a typical ER mTBI population. This selection leads to a higher percentage of CT-positive patients mainly due to the exclusion of CT-negative patients i.e. mTBI patients with GCS 15 and no additional clinical symptom. Further limitation was the heterogeneity of the cohorts, due to the differences in sample collection (serum and plasma), and the use of different immunoassays. Moreover, after stratifying by age, the remaining sample size was small, and these results should be verified and validated in a larger cohort.

## Conclusion

The present prospective multicentre study compared the measurement of levels of heart fatty-acid binding protein and S100B as potential tools to aid decision-making when clinicians need to objectively evaluate whether mTBI patients can be discharged without undergoing a CT scan. S100B performed better for older patients (≥ 65 years old) and H-FABP performed better for younger ones (< 65 years old). However, individual performance over the entire population revealed that H-FABP was an interesting protein biomarker for differentiating between CT-positive and CT-negative patients, within both 6 h and 24 h of trauma onset.

## Supporting information

S1 Table Characteristics, ≤6h post trauma, of the mTBI patients from Barcelona. (DOCX)

S2 Table Characteristics, ≤6h post trauma, of the mTBI patients from Seville. (DOCX)

S3 Table Characteristics, ≤6h post trauma, of the mTBI patients from Geneva. (DOCX)

S4 Table Characteristics of all the mTBI patients, t < 24 h after trauma onset. (DOCX)

S5 Table When sensitivity was fixed at 100%, the best performance ranged between 90%–100%, at S100Bs cut-off 0.1 μg/L and corresponding NPV and PPV. The results include all patients, t < 24 h after trauma onset.
(DOCX)

S1 Dataset All patient data. (XLSX)
