# Supplementary material for: H-FABP: A new biomarker to differentiate between CT-positive and CT-negative patients with mild traumatic brain injury
Source: PLoS One. 2017 Apr 18;12(4):e0175572. doi: 10.1371/journal.pone.0175572 (PMC5395174; doi:10.1371/journal.pone.0175572)
Supplement: S1 Table — (DOCX) [file pone.0175572.s001.docx]

| **S1 Table.** Characteristics, ≤6h post trauma, of the mTBI patients from Barcelona. | | | |
| --- | --- | --- | --- |
|  | **CT -** | **CT +** | **p-value**^†^ |
|  |  |  |  |
| **CT-scan**, n (%) | 29 (74) | 10 (26) |  |
| **Time trauma to blood** (min) |  |  | 0.558 |
| Mean (SD) | 123 (87) | 234 (83) |  |
| Median (min.-max.) | 230 (35-360) | 248 (55-335) |  |
| **Age**, mean (SD) | 46 (16) | 60 (23) | 0.055 |
| **Male**, n (%) | 19 (66) | 8 (80) | 0.332 |
| **Symtoms**, y (%) |  |  |  |
| Amnesia | 16 (55) | 2 (20) | 0.155 |
| LOC | 20 (69) | 4 (40) | 0.440 |
| Nausea/Vomits | 4 (14) | 2 (20) | 0.490 |
| Headache | 4 (14) | 0 (0) | 0.289 |
| Equilibrium impairment | 2 (7) | 0 (0) | 0.548 |
| **Mechanism of Injury**, n (%) |  |  |  |
| Traffic accident | 6 (21) | 2 (20) | 0.671 |
| Fall | 1 (3) | 0 (0) | 0.744 |
| Assult | 10 (35) | 2 (20) | 0.332 |
| Sports | 1 (3) | 1 (10) | 0.452 |
| Others | 11 (38) | 5 (50) | 0.380 |
| NA |  |  |  |
| **Isolated brain trauma**, y (%) | 11 (38) | 8 (80) | **0.026** |
| NA |  |  |  |
| ^†^ Chi-square test or Fisher’s exact test | | | |
| ^‡^ Mann-Whitney U-test. | | | |
| NA: not available | | | |
