# Supplementary material for: H-FABP: A new biomarker to differentiate between CT-positive and CT-negative patients with mild traumatic brain injury
Source: PLoS One. 2017 Apr 18;12(4):e0175572. doi: 10.1371/journal.pone.0175572 (PMC5395174; doi:10.1371/journal.pone.0175572)
Supplement: S2 Table — (DOCX) [file pone.0175572.s002.docx]

| **S2 Table.** Characteristics, ≤6h post trauma, of the mTBI patients from Seville. | | | |
| --- | --- | --- | --- |
|  | **CT -** | **CT +** | **p-value**^†^ |
|  |  |  |  |
| **CT-scan**, n (%) | 63 (89) | 8 (11) |  |
| **Time trauma to blood** (min) |  |  | 0.507 |
| Mean (SD) | 195 (81) | 209 (85) |  |
| Median (min.-max.) | 205 (50-360) | 245 (45-310) |  |
| **Age**, mean (SD) | 42 (20) | 50 (28) | 0.604 |
| **Male**, n (%) | 46 (73) | 7 (88) | 0.344 |
| **Symtoms**, y (%) |  |  |  |
| Amnesia | 39 (62) | 7 (88) | 0.150 |
| LOC | 51 (81) | 7 (88) | 0.548 |
| Nausea/Vomits | 23 (37) | 5 (63) | 0.151 |
| Headache | 45 (71) | 5 (63) | 0.439 |
| Equilibrium impairment | 0 (0) | 0 (0) |  |
| **Mechanism of Injury**, n (%) |  |  |  |
| Traffic accident | 24 (38) | 2 (25) | 0.380 |
| Fall | 25 (40) | 3 (38) | 0.611 |
| Assult | 6 (10) | 2 (25) | 0.220 |
| Sports | 0 (0) | 0 (0) |  |
| Others | 7 (11) | 1 (13) | 0.636 |
| NA | 1 (2) |  |  |
| **Isolated brain trauma**, y (%) | 48 (76) | 7 (88) | 0.420 |
| NA |  |  |  |
| ^†^ Chi-square test or Fisher’s exact test | | | |
| ^‡^ Mann-Whitney U-test. | | | |
| NA: not available | | | |
