# Supplementary material for: H-FABP: A new biomarker to differentiate between CT-positive and CT-negative patients with mild traumatic brain injury
Source: PLoS One. 2017 Apr 18;12(4):e0175572. doi: 10.1371/journal.pone.0175572 (PMC5395174; doi:10.1371/journal.pone.0175572)
Supplement: S3 Table — (DOCX) [file pone.0175572.s003.docx]

| **S3 Table.** Characteristics, ≤6h post trauma, of the mTBI patients from Geneva. | | | |
| --- | --- | --- | --- |
|  | **CT -** | **CT +** | **p-value**^†^ |
|  |  |  |  |
| **CT-scan**, n (%) | 48 (77) | 14 (23) |  |
| **Time trauma to blood** (min) |  |  | 0.115 |
| Mean (SD) | 195 (92) | 156 (102) |  |
| Median (min.-max.) | 193 (40-360) | 135 (40-360) |  |
| **Age**, mean (SD) | 50 (23) | 68 (23) | **0.013** |
| **Male**, n (%) | 36 (75) | 8 (57) | 0.168 |
| **Symtoms**, y (%) |  |  |  |
| Amnesia | 29 (60) | 9 (64) | 0.794 |
| LOC | 42 (88) | 14 (100) | 0.200 |
| Nausea/Vomits | 4 (8) | 2 (14) | 0.410 |
| Headache | 10 (21) | 3 (21) | 0.612 |
| Equilibrium impairment | 0 (0) | 0 (0) |  |
| **Mechanism of Injury**, n (%) |  |  |  |
| Traffic accident | 6 (13) | 6 (43) | **0.020** |
| Fall | 26 (54) | 7 (50) | 0.783 |
| Assult | 9 (19) | 1 (7) | 0.279 |
| Sports | 3 (6) | 0 (0) | 0.457 |
| Others | 1 (2) | 0 (0) | 0.774 |
| NA | 3 (6) |  |  |
| **Isolated brain trauma**, y (%) | 41 (87) | 9 (64) | 0.064 |
| NA |  |  |  |
| ^†^ Chi-square test or Fisher’s exact test | | | |
| ^‡^ Mann-Whitney U-test. | | | |
| NA: not available | | | |
