# Supplementary material for: H-FABP: A new biomarker to differentiate between CT-positive and CT-negative patients with mild traumatic brain injury
Source: PLoS One. 2017 Apr 18;12(4):e0175572. doi: 10.1371/journal.pone.0175572 (PMC5395174; doi:10.1371/journal.pone.0175572)
Supplement: S4 Table — (DOCX) [file pone.0175572.s004.docx]

| **S4 Table. Characteristics of all the mTBI patients, t < 24 h after trauma onset.** | | | |
| --- | --- | --- | --- |
|  | **All** | **CT -** | **CT +** |
|  |  |  |  |
| **CT-scan**, n (%) | 261 | 220 (84) | 41 (16) |
| **Trauma to blood sample** (min) |  |  |  |
| Mean (SD) | 250 (172) | 244 (156) | 274 (229) |
| Median (min.–max.) | 224 (35–1020) | 220 (35–990) | 230 (40–1020) |
| **Age**, mean (SD) | 50 (23) | 47 (22) | 63 (25) |
| **Male**, n (%) | 167 (64) | 142 (65) | 25 (61) |
| **Symtoms**, y (%) |  |  |  |
| Amnesia | 160 (61) | 129 (59) | 31 (76) |
| LOC | 196 (75) | 164 (75) | 32 (78) |
| Nausea/vomiting | 73 (28) | 61 (28) | 12 (29) |
| Headache | 112 (43) | 102 (46) | 10 (24) |
| Impaired equilibrium | 3 (1) | 2 (1) | 1 (2) |
| **Isolated brain trauma**, y (%) | 183 (70) | 154 (70) | 29 (71) |
| NA | 2 | 2 |  |
| NA: not available |  |  |  |
