# Supplementary material for: H-FABP: A new biomarker to differentiate between CT-positive and CT-negative patients with mild traumatic brain injury
Source: PLoS One. 2017 Apr 18;12(4):e0175572. doi: 10.1371/journal.pone.0175572 (PMC5395174; doi:10.1371/journal.pone.0175572)
Supplement: S5 Table — The results include all patients, t < 24 h after trauma onset. (DOCX) [file pone.0175572.s005.docx]

| **S5 Table. When sensitivity was fixed at 100%, the best performance ranged between 90%–100%, at S100Bs cut-off 0.1 µg/L and corresponding NPV and PPV. The results include all patients, t < 24 h after trauma onset.** | | | | | |
| --- | --- | --- | --- | --- | --- |
| **Protein** | **Cut-off** | **SE % (95% CI)** | **SP % (95% CI)** | **NPV** | **PPV** |
| **S100B** | 0.042 | 100 (100–100) | 5.9 (3.2–9.1) | 100 | 16.5 |
|  | 0.072 | 92.7 (82.9–100) | 19.5 (14.6–25.0) | 93.5 | 17.7 |
|  | 0.1 | 85.4 (73.2–95.1) | 39.6 (33.2–45.9) | 93.6 | 20.9 |
| **H-FABP** | 2.378 | 100 (100–100) | 25.5 (20.0–31.4) | 100 | 20 |
|  | 2.636 | 97.6 (92.7–100) | 30.5 (24.6–36.8) | 98.6 | 20.7 |
| SE, sensitivity; SP, specificity; NPV, negative predictive value; PPV, positive predictive value | | | | | |
